# Exploring the landscape of psychological threat: A cartography of threats and threat responses

**DOI:** 10.1111/spc3.12588

**Published:** 2021-02-26

**Authors:** Stefan Reiss, Eline Leen‐Thomele, Johannes Klackl, Eva Jonas

**Affiliations:** ^1^ Department of Psychology University of Salzburg Salzburg Austria

## Abstract

Over the recent years, research in the field of threat and defense has accumulated evidence on how encounters with various psychological threats influence human behavior, cognition, motivation, affect, and health. Unifying different theoretical threat models, the General Process Model of Threat and Defense claims that different threatening concerns have a similar underlying dynamic. Some years after the publication of this theory, we deem it important to take a comparative look at psychological threat, comparing threats regarding their properties and outcomes on personal and social level. As potential dimensions to describe psychological threats, we discuss the existential nature of concerns, phenomenological worlds involved, and thwarted needs in threat encounters. We also discuss data‐driven approaches to threat classifications, describing first empirical efforts to create threat taxonomies, and suggest directions for future research. This research will enhance our understanding of threat dynamics, and will help us make stronger, more clear‐cut assumptions about human behavior upon experiencing threat.

## 1 INTRODUCTION

Creating maps is an academic hobby: researchers across virtually all disciplines of science create classifications and taxonomies to be mapped (Brunn & Dodge, 2017b). Maps help to simplify complex areas of research, summarize findings, and facilitate creating plausible models of reality—they assist in navigating unknown territories. This goes beyond geographical maps of countries and continents: we map stars in the universe, musical genres, cultural practices, and art styles. We even map psychological functions to brain mechanisms and structures (for an overview, see Brunn & Dodge, 2017a).

A meaningful categorization of psychological threats and defenses can be beneficial both for researchers in the field of threat and defense, and laypeople. For researchers, theoretically founded and empirically testable assumptions of dimensionality across psychological threat can improve our understanding of threat‐and‐defense processes. Ultimately, they can help to make better predictions on how individuals react to specific psychologically threatening situations and create interventions to re‐route defenses into more beneficial paths for individuals and society. For laypeople, an overview of what defines a psychological threat, how threats influence us, and how we can appropriately deal with the arising unpleasantness, may nudge us to think and talk more about these processes. We need to reflect more on how threat affects interpersonal and intergroup relations across societies. Why do we become prone to cultural worldview defense, ethnocentrism, or zealous nationalism when feeling threatened? What causes us to confront strangers who hold conflicting political attitudes? Why do we try to rationalize or blame others instead of acting against climate change? In which circumstances will individuals try to resolve the cause of an issue instead of reacting defensively, possibly perpetuating conflicts (Jonas & Fritsche, 2013; Reiss & Jonas, 2019)?

We aim to organize threats and defenses and create a taxonomy. The challenge at hand is that before mapping we first need to define the dimensions that can organize threats. In geographical maps, spatial information can (with more or less ease) be projected onto defined values of geographical latitude and longitude. In the realm of threat and defense research, however, such dimensions are not yet defined, and thus warrant discussion. To find some orientation, we build on previous theoretical approaches to psychological threat and defense that may help to better understand and explain human reactions to threat. While the literature has provided a theoretical scaffolding to categorize defenses against psychological threats, there have been no attempts to do the same for the threats themselves. We suggest distinctions between existential and situational threats, between personal, social, and environmental threats, and in respect to thwarted psychological needs. Potential empirically measurable dimensions can be affective, motivational, behavioral, and cognitive reactions to psychological threat. From there, we will discuss first empirical investigations on the dimensionality of threat and the potential paths for future research.

## 2 THREAT AND DEFENSE—A GENERAL PROCESS MODEL

Since the late 1980s, the threat and defense literature has accumulated a vast amount of research on how psychological threat influences human physiology, affect, motivation, and behavior, both on a personal and societal level. This research suggests that the experience of psychological threats such as uncontrollability, uncertainty, or the awareness of one's mortality can foster negative attitudes towards outgroups, intergroup bias, and the defense of cultural worldviews. For example, climate change threat has shown to increase authoritarian tendencies (Fritsche, Cohrs, Kessler, & Bauer, 2012), confrontation with one's mortality has increased religious zeal (McGregor, Nash, & Prentice, 2010) and nationalism (Pyszczynski et al., 2006). Many models and theoretical approaches regarding psychological threats and defenses show substantial overlap (e.g., cognitive dissonance theory, Festinger, 1957; self‐affirmation theory, Steele, 1988; terror management theory, Greenberg et al., 1990; meaning maintenance model, Heine, Proulx, & Vohs, 2006). Between theories, there are conceptual similarities in what constitutes a threat, how individuals instantaneously react to it, and how they manage to leave this immediately activated state. Jonas and colleagues (Jonas et al., 2014) formulated a unifying model of threat and defense that intents to explain and predict individuals' responses to threats.

This model, termed *General Process Model of Threat and Defense* (GPM; Jonas et al., 2014), postulates that threat is the experience of discrepancy between the situation, a personal current cognitive focus, or current personal motives (Jonas & Mühlberger, 2017). People then usually experience anxiety, arousal, and attentional vigilance (for a more in‐depth discussion of the biological processes, see Gray & McNaughton, 2000; McNaughton & Corr, 2004). In cases where humans cannot resolve the source of the threat (e.g., they cannot achieve literal immortality), they need to deal with the anxiety in a different way: by avoiding threat‐related thoughts, a strategy termed *proximal defense*. After a delay—when thoughts of the threat have been removed from conscience—a second set of defense mechanisms follows, the so‐called *distal defense*s. If a concrete resolution of the threat is easily available, people can directly approach this resolution and try to mitigate the anxious state. However, if a solution is not easily available, people might turn to strategies that indirectly solve the threat or that are palliative. For instance, after confronting people with the prospect of their own mortality, they defend their own culture more strongly than people in a nonthreatening control condition (Burke, Martens, & Faucher, 2010; Pyszczynski, Solomon, & Greenberg, 2015). However, defending one's own culture is not the only possible behavioral response to psychological threat.

**FIGURE 1:**
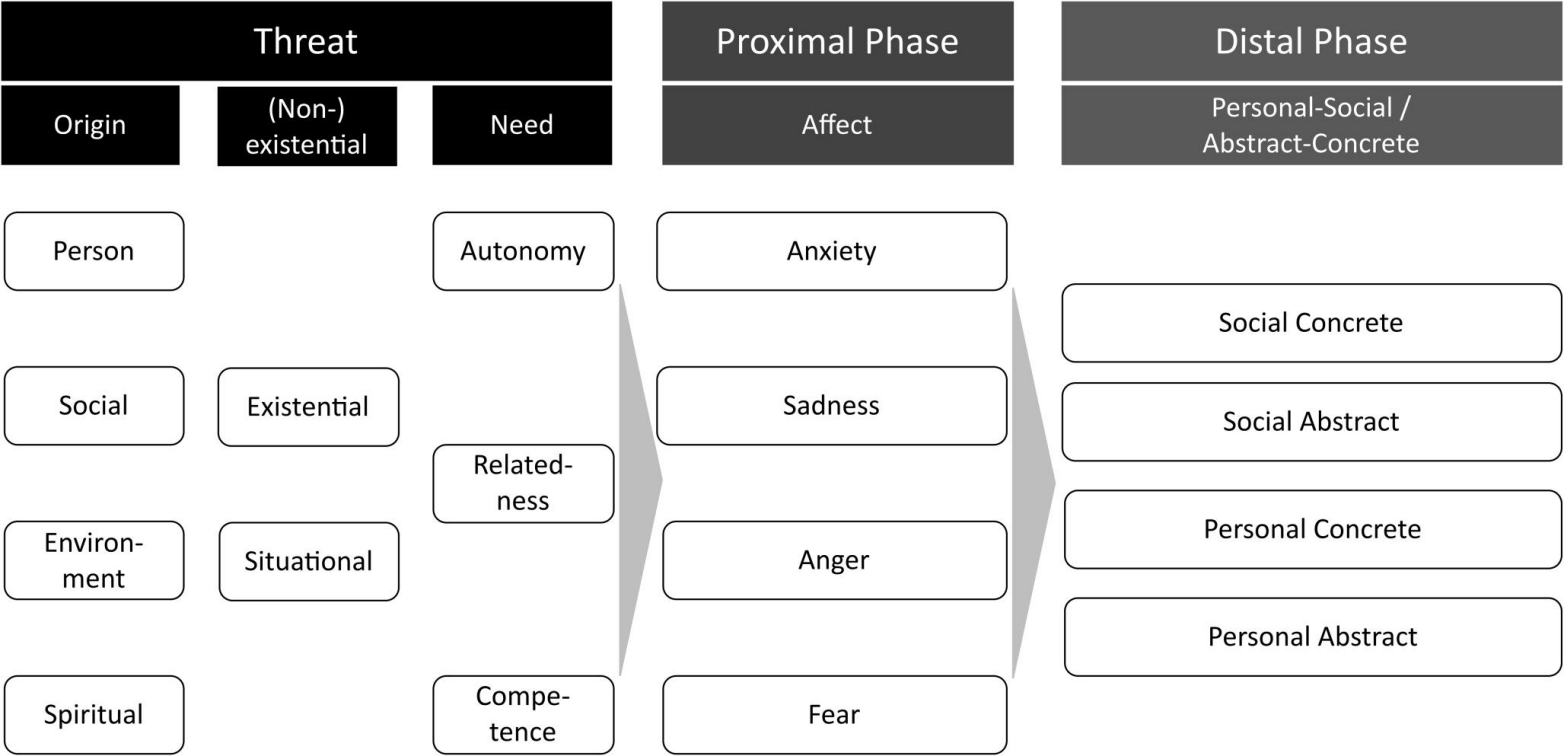
Suggested dimensions to map threat‐and‐defense processes, based on the general process model of threat and defense (Jonas et al., 2014)

The GPM has made explicit suggestions on the dimensions along which to map defenses, namely abstract versus concrete and personal versus social (Jonas et al., 2014). However, dimensions of threats have not been proposed or investigated. We will first discuss potential dimensions to categorize psychological threats, and subsequently the defense taxonomy provided by the GPM (Jonas et al., 2014). The proposed dimensions are illustrated in Figure 1 and exemplary categorization  can be found in Table 1.

**TABLE 1:** Exemplary categorization of psychological threat, according to the proposed dimensions

Threat	Phenomenological origin	Existential versus Situational	Need	Affect	(Main) defense category	Exemplary reference
Mortality	Personal	Existential	Autonomy/Competence/Relatedness	Anxiety, sadness, sorrow	Personal abstract/Social abstract/Social concrete	Pyszczynski et al. (2004); Florian & Mikulincer (1998)
Isolation	Social	Existential	Relatedness	Anxiety, sadness	Social concrete/Social abstract	Pinel et al. (2004)
Uncertainty	Personal/Environment	Existential	Competence	Anxiety	Social abstract	Hogg et al. (2007)
Meaning	Environment	Existential	Competence	Anxiety	Personal abstract/Social abstract	Proulx and Inzlicht (2012)
Freedom of action	Personal/Environment	Situational	Autonomy	Hostility, nervousness, anger	Personal concrete	Sittenthaler et al. (2015)
Social exclusion	Social	Situational	Relatedness	Sadness, anxiety	Social concrete	Maner et al. (2007)
Relationship threat	Social	Situational	Relatedness	Anxiety	Personal concrete	McGregor et al. (2010)
COVID‐19 pandemic	Environment/Social	Existential and situational	Autonomy/Competence/Relatedness	Anxiety, hostility, sorrow	Personal concrete/Personal abstract/Social concrete/Social abstract	Jutzi et al. (2020); Reiss et al. (2020a)

## 3 TAXONOMY OF THREATS

When trying to categorize and compare threats, there are two aspects of threats that should be distinguished: descriptive information about *properties* of threats and *responses* to the threats (i.e., immediate affective states and subsequent defensive strategies). As a first step, we will consider which dimensions researchers have used so far to explain where threat arises, which reactions it causes immediately, and how individuals defend against it.

### 3.1 Concreteness and distance

We can approach mapping psychological threat by first describing them, using objective properties inherent to various psychological threats. For instance, is a threat more abstract or concrete, and is it temporally close or distant? To give some examples, mortality salience is usually defined as a temporally and spatially distant threat, as the time and place of death is unknown to most people. Expectancy violations such as nonsensical text passages or reverse‐colored playing cards on the other hand are usually temporally and spatially close because they happen in the here and now (Proulx & Major, 2013; Reiss, Klackl, Proulx, & Jonas, 2019b). Freedom restrictions are mostly construed as concrete, real‐life situations (Sittenthaler, Steindl, & Jonas, 2015; Steindl & Jonas, 2015). Recalling specific events in which one has absolutely no control (Kay, Gaucher, Napier, Callan, & Laurin, 2008) may be more or less temporally and spatially distant, but naturally, these events have happened in the past.

### 3.2 Phenomenological worlds of threat

Threats may be meaningfully categorized according to the phenomenological world from which they originate. To analyze dimensions of threat, we first need to set the place where the experience of threats and the defensive reactions to them take place. According to the phenomenological line of thinking, human life takes place in four worlds that are inherently interwoven: our physical environment; the social world we share with others; private and inner experiences; and the connection to spiritual entities and processes (Binswanger, 1958; Pyszczynski, Sullivan, & Greenberg, 2014). This results in various questions we can ask to understand threats: where does a certain threat originate? Which phenomenological realm is compromised? Where do individuals turn their attentional focus? Under which circumstances do people turn to social identities, worldviews, or ethnocentrism to defend themselves?

Understanding that life takes places in these phenomenological realms simultaneously, can we assume that psychological threats can compromise an individual's relation to these realms? For instance, existential isolation is threatening one's social relatedness to others. The current coronavirus pandemic has been proposed as a *super‐threat* combining concerns about our own mortality, meaninglessness, uncertainty, and a lack of personal control (Jutzi, Willardt, Schmid, & Jonas, 2020), threatening one's connections to the physical and social environment. Table 1 may serve as a basis for discussion but does not claim to be definitive. We acknowledge that these phenomenological worlds are inherently interwoven, even more so when considering real‐world super‐threats compared to theoretically derived existential concerns. However, we do think that most threats are primarily linked to one of the worlds. The phenomenological worlds help us to locate personal, social, and environmental aspects of being.

### 3.3 Existential versus nonexistential threats

One basic distinction between psychological threats is whether they are *existential* or not. The focus of threat research over the last years has been on *existential* threats. Greenberg and colleagues wrote an entire book about the experimental investigation of existential concerns (Greenberg, Koole, & Pyszczynski, 2004). Based on Yalom's existential psychotherapy (Yalom, 1980), they formulated existential concerns influencing humans' behavior, intentions, motivation, and affect. According to Yalom, the four main concerns of existential psychotherapy are death, freedom, isolation, and meaninglessness. However, he emphasized that the label *existential* “defies succinct definition” (Yalom, 1980, p. 5). More recently, other authors have proposed more precise definitions of existential threats. Sullivan and colleagues referred to philosopher Paul Tillich in defining four key aspects of existential threat: *anxiety*, *awareness*, *nonbeing*, and *existential*. Briefly explained, a threat is existential in nature when it causes stimulus‐unspecific negative affect (i.e., *anxiety,* not fear), and involves some degree of self‐consciousness (awareness), conveys the potential of one's own *nonbeing* in either of the phenomenological worlds (Sullivan, Landau, & Kay, 2012). Experimental existential psychologists listed the “big five” of existential concerns: death, isolation, identity, freedom, and meaning (Koole, Greenberg, & Pyszczynski, 2006).

This definition suggests that the big five existential threats have quite concrete situational, nonexistential counterparts: Situational social exclusion is the nonexistential counterpart of existential isolation; reactance as a reaction against concrete restrictions of freedom of action (Torrance & Brehm, 1968) is the counterpart of the existential concern of freedom (better referred to as responsibility in Sartre's terms). Inconsistencies between related cognitive elements (termed *cognitive dissonance*, Festinger, 1957) are a nonexistential instance of existential meaning concerns (i.e., that life has no meaning), situational uncertainty is the concrete counterpart of existential uncertainty about one's self, and concrete threats to one's life are instances of existential concerns of mortality.

To summarize, in addition to the experiential world thwarted by a threat, we can distinguish existential and nonexistential psychological concerns. Literally, these existential givens relate to human existence and how we connect to the world around us. As Table 1 shows, this categorization allows to categorize threats in more detail. To further disentangle threats, we should also consider the needs in jeopardy.

### 3.4 Threatened psychological needs

According to GPM, threat is defined by the experience of a discrepancy, be that perceptual, epistemic, or motivational. Frustrations of basic psychological needs are the essence of discrepancies in threats and provide motivational fuel. While most individuals can deal with short‐term need frustrations, prolonged need frustration and anxiety can have detrimental effects such as decreased life satisfaction, increased negative affect (Routledge et al., 2010), decreased pursuit of everyday goals (Pyszczynski, Greenberg, & Solomon, 1999), or even posttraumatic stress (Abdollahi, Pyszczynski, Maxfield, & Luszczynska, 2011; Pyszczynski & Taylor, 2016).

According to self‐determination theory (Deci & Ryan, 2000; Ryan & Deci, 2000), humans have three basic psychological needs: autonomy, competence, and relatedness. Autonomy describes the need to perceive one's life as meaningful and own actions as having purpose, to be self‐governing and true to one's values. Competence describes feeling knowledgeable or experiencing predictability and control in their environment. Relatedness is the need to feel socially embedded. These basic needs function like vitamins in human motivation, that means only when all three needs are fulfilled to a minimal extent, goal‐pursuit can take place. In our regular lives, there will invariably be situations that threaten immediate need fulfillment, if just for a little while. Ongoing confrontations with frustrated need fulfilment can lead to apathy, amotivation, vulnerabilities for psychopathology, or negative affect (Vansteenkiste & Ryan, 2013). Thus, one powerful way of reducing discrepancies is through fulfilling psychological needs. Empirical evidence has shown that the frustration of psychological needs may foster biased information processing and intergroup biases (Lueders, Prentice, & Jonas, 2019). Thwarted need fulfillment may thus be a dimension for mapping threats linked to the phenomenological worlds. To be more precise, not only should we investigate *to what extent* needs are threatened, but also *which needs* these are. Do concerns that threaten relatedness, such as isolation, differ from concerns that primarily threaten autonomy, such as freedom?

We propose that the phenomenology of threat, that is, whether a threat is experienced as personal, social, or environmental, may specifically be related to the fulfilment of specific psychological needs: Personal threats are most likely associated with autonomy need fulfilment, for instance the responsibility of choice or self‐uncertainty. Threats originating from the social world, such as isolation, are tied to relatedness needs. And threats from the physical environment, such as meaning threats or surprise, are related to competence needs. These assumptions still need to be tested empirically but might hold up well in a study set up to systematically combine phenomenology and needs. For hypothesized associations of psychological needs to exemplary threats, refer to Table 1.

Now that we have set up an atlas of phenomenological worlds and populated it with threats, we can locate the origins of threats, their contact points to experiential worlds, and the worlds individuals use to defend themselves. In a next step, we will focus on the outcomes of threat experiences.

## 4 TAXONOMY OF THREAT RESPONSES

### 4.1 Immediate responses as dimensions for mapping

Moving on from descriptors of threat sources, another way to approach mapping is from the perspective of individuals' responses to threat experience. For example, while mortality salience typically elicits anxiety, sadness, and sorrow (Kastenbaum & Heflick, 2011; Lambert et al., 2014; Reiss, Klackl, & Jonas, 2021), social exclusion typically elicits pain and sadness (Hawkley, Williams, & Cacioppo, 2011, see also Table 1).

There is little research comparing the (neuro‐)physiological responses to different threat manipulations. However, functional neuroimaging (fMRI) has shown that apart from large overlaps in activated brain regions the salience of mortality and uncontrollability caused different activation patterns: mortality salience (vs. uncontrollability) increased activation in the posterior cingulate cortex, an area considered important for conflict detection. The right anterior insula, an area associated with introspection, was less activated following a mortality (vs. uncontrollability) prime (Klackl, Jonas, & Fritsche, 2017). In terms of peripheral physiology, existential threats were mostly identical to a (nonexistential) social‐evaluative threat condition regarding heart rate, skin conductance, and other physiological indicators of arousal (Poppelaars, Klackl, Scheepers, Mühlberger, & Jonas, 2020). However, the subjective experience of negative affect was higher following existential threat salience (mortality, uncontrollability, uncertainty) compared to nonexistential (social‐evaluative) threat. This suggests again that while different threats share substantial commonalities there are also differences in physiology and experience that make threats unique and distinguishable.

### 4.2 Distal threat responses

The categorization of delayed (i.e., distal) defense mechanisms and strategies serves a heuristic and explanatory function. It helps us to better understand and break down the sheer uncountable amount of defense strategies people presumably make use of when confronted with psychological threat. Depending on the theoretical framework, defenses can have varying purposes. For instance, they may serve to reinstate a general, global sense of control (Fritsche, Jonas, & Fankhänel, 2008; Fritsche et al., 2017), a sense of meaning and predictability in the environment (Heine et al., 2006; Proulx, 2012; Proulx & Inzlicht, 2012), to regain self‐esteem (Pyszczynski et al., 1999), or to enable goal pursuit (McGregor, Nash, Mann, & Phills, 2010; Nash, McGregor, & Prentice, 2011).

In the general process model (Jonas et al., 2014), distal defenses are conceptualized in two dimensions: abstract versus concrete and personal versus social. In broad strokes, the model differentiates between value‐related abstract defenses and concrete action‐related defenses. For instance, individuals may defend against a threat by trying to find relief in social relations (*concrete social*, e.g., Maner, DeWall, Baumeister, & Schaller, 2007), or commit to tangible personal rewards (*concrete personal*, e.g., pursuing personal projects; McGregor et al., 2010). Examples for abstract defenses are attempts to strive for intangible goals (*abstract personal*, e.g., self‐esteem; Pyszczynski, Greenberg, Solomon, Arndt, & Schimel, 2004) or group‐based control (*abstract social*; Fritsche et al., 2013). This way, we can investigate overlaps in phenomenological pathways of threats: following the source of a threat, which need is under attack, and what defensive focus individuals primarily take.

So far, we have discussed the general process model of psychological threat and defense, and potential dimensions that might be helpful in creating a taxonomy for threats. We can use descriptions of the phenomenology and existential nature of a threat as well as threatened needs to categorize the properties of a threat itself, and immediate responses and delayed defensive strategies to threat encounters to describe threat experiences. In our lab, we have started attempts to empirically investigate the dimensionality of threats and defense. We will briefly report these attempts and the first resulting insights provided by them.

## 5 EMPIRICAL TESTS OF THREAT TAXONOMIES

As a first data‐driven approach, we presented seven threat manipulations (mortality salience, uncontrollability, uncertainty, freedom restriction, identity, meaning threat, and social‐evaluative threat) and a neutral TV salience (as a no‐threat control condition) to a sample of 239 students. We asked them to write down their thoughts and emotions when thinking about the topics (e.g., “Please think about the emotions that the thought of your own death causes in you. Please also think about what will happen to you when you die and when you are dead”). They were then asked to rate the threats on multiple dimensions, like concrete‐abstract, time (past‐present‐future), and state affects such as *anxious*, *uncertain*, and *inhibited* (Reiss, Klackl, & Jonas, 2020b; materials and data available; Reiss et al., 2021). This allowed us to compare the threat scenarios against a neutral no‐threat condition (TV) and against each other.

**FIGURE 2:**
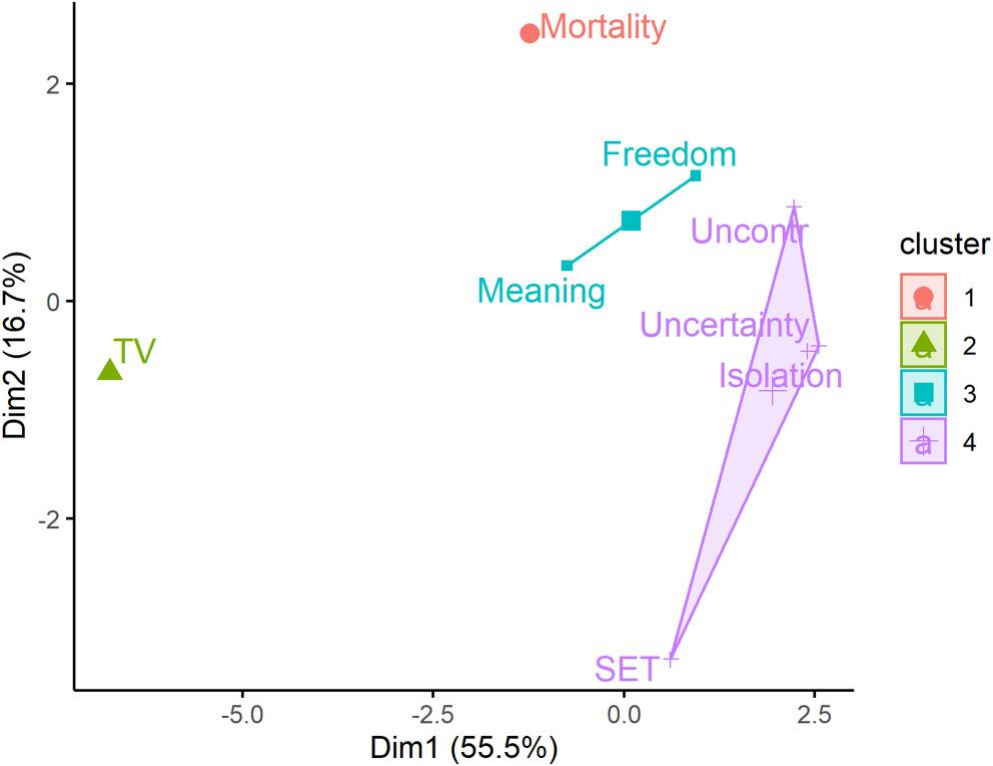
Four‐cluster solution of k‐means threat clustering. For illustrative purposes, the solution is plotted in two‐dimensional space using principal component analysis. Data from manuscript currently in preparation (*n* = 239; Reiss, Klackl & Jonas, 2021). SET, social‐evaluative threat; Uncontr, uncontrollability threat

Based on the measured threat features, a clustering algorithm suggested a four‐cluster solution to categorize the eight tested manipulations. As expected, TV salience as a neutral control was identified as a unique cluster, suggesting a neutral control was consistently distinguishable from all threat inductions. Mortality salience formed another single‐threat cluster. Freedom and meaning threat were identified as a two‐threat cluster. Isolation, social‐evaluative threat, uncertainty, and uncontrollability formed the biggest cluster, suggesting that they had overlapping values on the classifying features (Figure 2). The results suggest that TV salience as control is substantially different from all threat inductions, and that mortality salience takes a unique position among the presented threat inductions. This is in accordance with threat theorists positioning mortality as a special threat among others (Greenberg et al., 1990), but does not necessarily imply that it is the *core threat* underlying all threat‐and‐defense processes. In this study, we looked mostly at various descriptive dimensions such as time, concreteness, or intensity. Still, we provided first evidence for dimensionality in psychological threats. We are planning further studies with the intent to replicate these findings, include *super‐threats* such as climate change or COVID‐19, and systematically assess the dimensions brought forward in this article.

**FIGURE 3:**
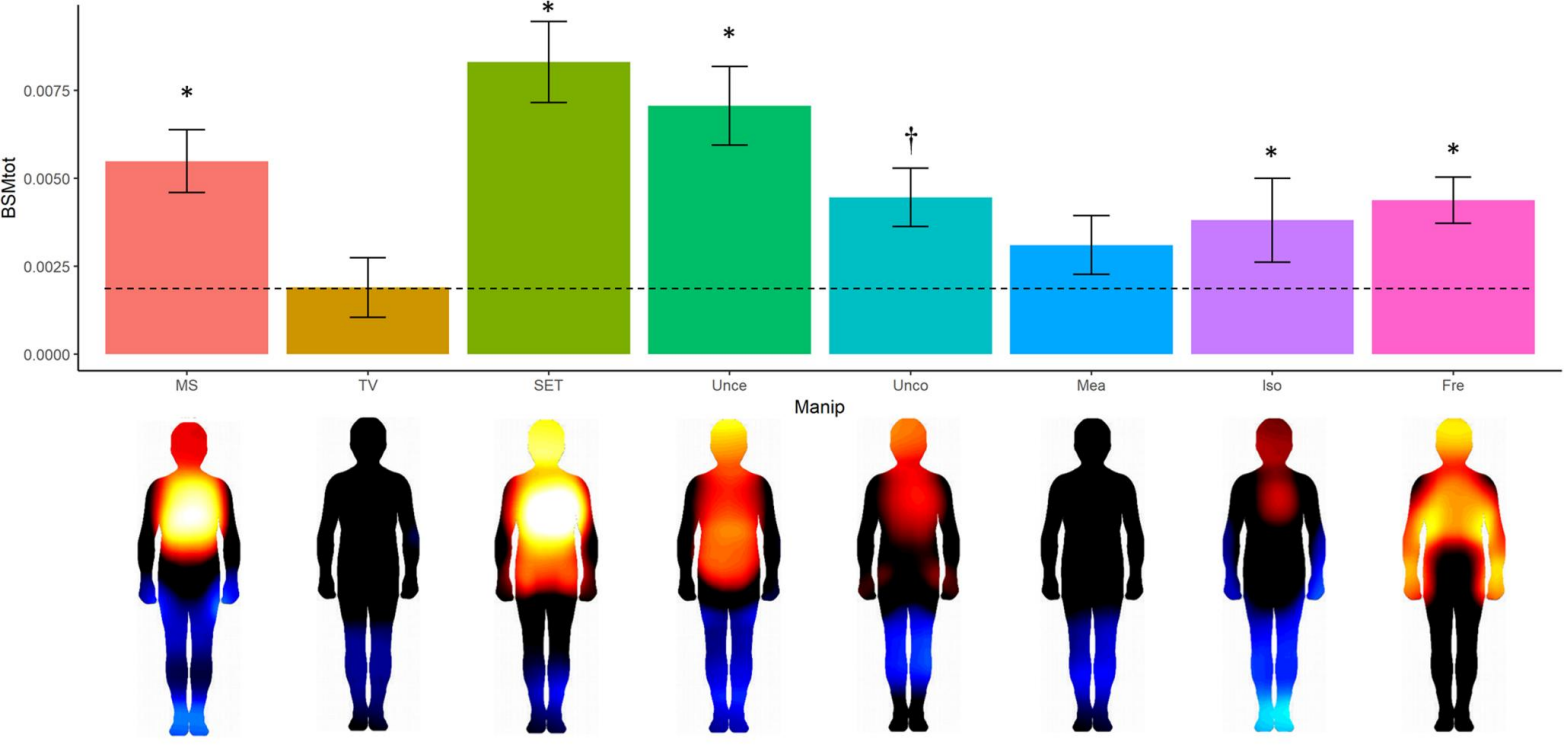
BSM in reaction to different threat salience manipulations. Values indicate mean absolute values of change in bodily arousal across the entire body. Statistical significance is indicated for comparisons versus TV salience (Tukey HSD corrected; *p < 0.001; ^†^0.05 < *p* < 0.10). Data and figure adapted with permission from Reiss et al. (2021). BSM, bodily sensation maps; Fre, freedom threat; Iso, isolation threat; Mea, meaning threat; MS, mortality salience; SET, social‐evaluative threat; TV, TV salience; Unce, uncertainty threat; Unco, uncontrollability

To gauge the effect of threat salience on experienced affect and arousal, we conducted another study. Here, we compared the effect of the same seven manipulations mentioned above and TV salience as control on a different measure: we employed bodily sensation maps to let participants (*n* = 92) indicate perceived increases and decreases in bodily activity on blank body silhouettes (Nummenmaa, Glerean, Hari, & Hietanen, 2014; Reiss, Agroskin, Klackl, McGregor, & Jonas, 2021; Reiss, Prentice, Schulte‐Cloos, & Jonas, 2019c). Results suggest that while virtually all manipulations elicited more bodily arousal across the entire body than the no‐threat TV salience, the locations of where this activity takes place is different (Figure 3). For instance, freedom threat lacks the deactivation in the leg region found in the other threat salience conditions; social‐evaluative threat elicits very strong bodily activation overall, and specifically in the head/face area; and isolation priming elicit a strong deactivation in the feet, but not much activation in the chest and head areas. Further analyses can be conducted here to classify and cluster threat indices for the different concerns. For now, these studies and analyses suggest that there are empirically measurable differences between the bodily experience of different psychological threats. So far, we cannot make predictions about subsequent defensive responses. However, we plan further mediational studies to investigate whether these experiential arousal states translate to subsequent defense strategies.

Moving on from threat descriptors and immediate affect and arousal, investigations of mediational effects of direct resolution and palliative efforts have also shown differences between threats. Both existential and nonexistential threats were shown to elicit anxious uncertainty. Palliation, that is, efforts aimed to reduce aversive arousal only helped to reduce anxiety following existential threat, but not situational threat. Thus, palliation seems to be more effective when coupled to the experience of existential threat. Direct resolution, in both conditions, was effective in re‐instating approach motivation, but did not significantly reduce anxiety (Stollberg, Klackl, & Jonas, 2020).

In sum, we have reported first examples of empirical attempts to investigate dimensionality of psychological threat in a data‐driven way. While overall we have found similar initial responses to various threats compared to a neutral control condition, there were substantial differences that differentiate threat manipulation. This pattern becomes even more complex when considering delayed reaction to threat,: while the aversiveness of existential concerns could be somewhat mitigated through palliative responses, this was not the case for non‐existential, situational threat.

The dimensions we have proposed in this paper are displayed in Table 1. The categorization in this table is based on previous studies. We think it will be valuable to systematically assess a psychological threat along the dimensions of phenomenological origin, its existential nature, thwarted psychological needs, the caused affective responses, and the likeliest defense strategy. Thus, we can disentangle the processes of threat and defense. We are confident that this approach will sharpen our conception of how different experiences of psychological threats may shape defensive responses in different ways. Using adequate sample sizes, the resulting patterns should inform us about the specific dynamics of threats as described by our taxonomy brought forward in this manuscript and potentially reveal starting points.

## 6 DISCUSSION AND OUTLOOK

In this manuscript, we revisited the current literature on psychological threat and defense, aiming to find clues for the categorization of psychological threat. Our goal is to move ongoing research towards a comprehensive understanding about which aspects of threats compel individuals to react in a group‐based, worldview‐related, *palliative* manner instead of approaching an issue with the intent of resolving it. Furthermore, we aim to better understand which defensive strategies can be helpful in relieving individual anxiety depending on the experienced threat. This understanding will hopefully guide the way to re‐route defenses in order to reduce group conflicts and societal issues driven by threat experience (Jonas & Fritsche, 2013; Motyl, Rothschild, & Pyszczynski, 2009; Pyszczynski, Motyl, & Abdollahi, 2009; Reiss & Jonas, 2019). Considering the GPM, a unifying model that emphasizes similarities in threat and defense processes, our approaches serve as a counterweight with the intent to disentangle the uniqueness for each threat. These need not be conflicting but serve their respective purposes. While former helps to explain the coarse processes shared across psychological threat experience regarding early and delayed responses to threat, the mapping approach tries to disentangle and understand what distinguishes the experience of one threat from another.

We have reported evidence that psychological threats can elicit broadly general, but individually different outcomes in affect, subjective arousal, and physiology. Furthermore, the effectiveness of resolution and palliation strategies in delayed defenses may be different for existential and nonexistential threats, respectively. These early results are promising that further experimental research may provide even more fine‐grained insights into these dynamics.

### 6.1 Future research: Toward a predictive model for threat and defense processes

Experimental threat research faces certain limitations in operationalization: normally, participants are presented one or very few different ways to defend themselves against (i.e., deal with) psychological threat. A dependent variable that shows effects is then assumed to be a defensive response. However, this approach has been criticized, comparing it to offering hungry participants only broccoli and then claiming broccoli is the best way to satisfy hunger (Galinsky, Whitson, Huang, & Rucker, 2012; Steele, 1988).

Some theoretical frameworks of threat and defense assume that threat compensations are fluid in their content. Thus, one manipulation could trigger different compensatory efforts, depending on context and individual predispositions (Proulx & Inzlicht, 2012). This poses a crucial challenge for the predictiveness of threat and defense models. In our opinion, this challenge can be tackled by systematically comparing threat manipulations regarding their descriptive dimensions, individuals' immediate reactions, and subsequent defensive responses. For instance, research has shown that both collective and individual threats can increase collective responses such as ethnocentrism and intergroup biases (Fritsche, Jonas, & Kessler, 2011)—but can we make informed prediction under which circumstances collective and individual threats lead to different outcomes?

This might allow us to find robust connections regarding aspects of psychological threat that precede certain defenses and allow to formulate targeted interventions. The experience of threat is highly subjective, thus, a purely mechanistic understanding of threat‐and‐defense processes is implausible. Our results so far suggest differences between threats, but so far these differences are not investigated enough to allow specific predictions on subsequent responses. However, with greater sample sizes and accumulating evidence over the course of multiple studies and empirical assessments, we hope to be able to find patterns in individual experiences of psychological threats.

In more concrete terms, future research should consider the following remarks. First, we need to consider individual experiences of threats. For instance, is COVID‐19 threat equally an uncertainty or a mortality concern across different individuals? How does this shape initial and delayed reactions? Furthermore, we need to systematically investigate individual defense strategies, both by providing an array of possible actions to handle anxious uncertainty, and by assessing what individuals would intuitively do in the respective situation. Employing such an inductive approach in combination with ratings of pre‐defined defensive strategies may reveal a lot about the individual level of threat compensation, but together inform about systematic patterns for different psychological threats or threat properties.

### 6.2 Practical implications

We think that the concepts brought forward in this article can also provide practical implications. First, a more clearly defined understanding of the nature of threats may help us to better understand which processes and mechanisms drive defensive responses. We have discussed findings that different threat manipulations generally induce anxiety and inhibition, but the effectiveness of resolution and palliation efforts in dealing with anxiety is different between existential and situational threats (Stollberg et al., 2020).

Facing psychological threats is inevitable: at some point in our lives, we must address uncomfortable facts, such as our own mortality. Recently, people all over the world had to face one of the worst pandemics in modern history, again forcing us to confront uncertainty, uncontrollability, and the potential of our own or loved one's demise (Jutzi et al., 2020; Tabri, Hollingshead, & Wohl, 2020). Making decisions with positive effects on a societal level is crucial: turning to intergroup biases and worldview bolstering might help individuals cope better with uncertainty and anxiety, but it will not reduce the issues at hand (or create other issues). Prosocial attitudes and compliance with restrictions will reduce threat for others (in addition to the self). When anxiety is high—especially during concrete *super threats* such as the COVID‐19 pandemic, we should face the threat and try to mitigate the real‐world consequences as much as possible.

The understanding of the intricacies of threat‐and‐defense processes may also be valuable to political decision‐makers. By understanding what drives instantaneous and delayed responses, and which threat experiences are more likely to lead to positive distal reactions, we can improve the communication of potentially worrying information without it leading to negative or inhibited responses by the recipients. Trying to mitigate negative defenses that increase intergroup biases and may perpetuate intergroup conflicts, information should be presented in a way that assists an individual to not be overwhelmed with negative affect leading to attentional avoidance of threat‐related thoughts and cognitions. Instead, they should face the issue and try to “deal with it”, that is, trying to resolve the issues at hand and not drifting to palliative behaviors and attitudes. For policymakers, this implies communicating a topic in a way that prevents individuals from avoiding the topic and engaging in unrelated, abstract defenses that make themselves feel better, but provide no sustainable solution for an issue. Following existential concerns, palliative efforts may prove helpful to deal with aversive arousal, however following concrete situational psychological threat, resolution efforts may provide the more effective route (Stollberg et al., 2020).

An important exemplary topic is the communication of climate change, a scientifically evidenced process that carries an ever‐increasing urgency to act. However, many people react to news about melting glaciers, rising sea levels, desertification of wide areas, deforestation of the amazon, and scarceness of resources with avoidance, indifference, or even active pollution. Thus, communication with overwhelmingly threatening information may lead individuals to abstract, threat‐unrelated defenses (Uhl, Jonas, & Klackl, 2016; Uhl, Klackl, Hansen, & Jonas, 2017). Instead, providing concrete guidelines for beneficial behavior and encouraging to face the threat should result in more positive outcomes.

For health practitioners, these insights might also be a helpful guidance. The understanding of the phenomenology and the frustrated needs in specific situations can improve the intervention for clients or patients dealing with anxiety. In some circumstances, trying to resolve a goal conflict at hand is a good way to re‐establish agency and approach, but sometimes relieving uncertainty through palliative efforts is just as, if not more viable for individuals.

### 6.3 Concluding thoughts

With this manuscript, we put forward a theoretically funded but tentative taxonomy of psychological threat and threat responses, and propositions for future empirical investigations in threat research (along with first empirical results). While we acknowledge the interrelatedness of psychological threats and the findings that have shown commonalities between threat manipulations, we argue that the distinctions between threats may be an important endeavor for future research. This will require systematic research of threat properties, individuals' perceptions of these threats, and both immediate and delayed reactions.

## CONFLICT OF INTEREST

The authors report no conflict of interest.
